# The effectiveness of a web-based decision aid for patients with hip osteoarthritis: study protocol for a randomized controlled trial

**DOI:** 10.1186/s13063-020-04661-z

**Published:** 2020-08-24

**Authors:** Lilisbeth Perestelo-Pérez, Yolanda Álvarez-Pérez, Amado Rivero-Santana, Vanesa Ramos-García, Andrea Duarte-Díaz, Alezandra Torres-Castaño, Ana Toledo-Chávarri, Mario Herrera-Perez, José Luis País-Brito, José Carlos del Castillo, José Ramón Vázquez, Carola Orrego, Pedro Serrano-Aguilar

**Affiliations:** 1 Evaluation Unit of the Canary Islands Health Service (SESCS), Camino Candelaria, s/n. 38109, El Rosario, S/C de Tenerife Spain; 2 Health Services Research on Chronic Patients Network (REDISSEC), Tenerife, Spain; 3 Center for Biomedical Research of the Canary Islands (CIBICAN), Tenerife, Spain; 4 Fundación Canaria Instituto de Investigación Sanitaria de Canarias (FIISC), Tenerife, Spain; 5 Hospital Universitario de Canarias (HUC), Tenerife, Spain; 6 Hospital San Juan de Dios de Santa Cruz de Tenerife (HSJD), Tenerife, Spain; 7 Gerencia de Atención Primaria de Tenerife del Servicio Canario de la Salud, Tenerife, Spain; 8 Avedis Donabedian Research Institute (FAD), Barcelona, Spain

**Keywords:** Hip osteoarthritis, Patient decision aids, Person-centered care, Shared decision-making, Web-based intervention, Randomized controlled trial

## Abstract

**Background:**

Osteoarthritis (OA) is a health condition sensitive to patient’s preferences and values regarding the benefits and risks of the different treatment options. In this sense, patient decision aids (PtDA) can play an important role in helping patients to incorporate their values, needs, and preferences into the decision-making process, thus improving person-centered care. Previous research has focused almost exclusively on knee OA, and therefore, the aim of this study is to develop and evaluate the effectiveness of a PtDA for patients with hip OA.

**Methods:**

The general design consists of two phases: (1) design a web-based PtDA for patients with hip OA, following the recommended procedures: systematic review of safety/effectiveness of treatments, and an iterative process of development with the help of an Advisory Committee composed of health professionals and patients, and (2) to evaluate the impact of the PtDA on hip OA patients’ decision-making process related with their treatment. For that aim, a multicenter randomized controlled trial will be carried out with 124 patients with hip OA in Tenerife (Spain) comparing intervention or usual care.

**Discussion:**

PtDAs have been recommended as a useful and effective resource for improving PCC in many health conditions. The intervention is intended to empower patients by fostering their active participation during the decision-making process about their treatment and by ensuring they make informed decisions congruent with their values and preferences. This study will contribute to the scientific knowledge about effectiveness of PtDAs in hip OA, in order to improve the quality of health care offered to these patients.

**Trial registration:**

ClinicalTrials.gov NCT04241978. Registered on 24 January 2020.

## Background

Osteoarthritis (OA) is one of the most prevalent chronic diseases [1], which affects more than 70% of people older than 50 years, and its prevalence clearly increases with age until 70 years old [2]. It can affect any joint, but preferably affects the knee, hands, hip, and spine [3]. Pain, stiffness, and loss of functionality caused by OA have an important impact in the quality of life of patients [4, 5]. Furthermore, OA represents a high economic burden for society, due to its direct and indirect health costs [6]. As a chronic disease, OA is associated with long-term multicomorbidity and polymedication.

Conservative therapeutic options for hip OA include exercise, weight loss, and walking aids, as well as drugs and corticosteroids injections in more advanced stages. When these options fail to improve symptoms, a surgical intervention called arthroplasty or joint replacement is recommended, with some variations depending on the damaged joint. This technique has been shown to be safe and effective [7], although like any other surgical procedure, it implies risks such as infection, thromboembolism, or the need for re-intervention due to problems in the prosthesis [8]. Indication for arthroplasty is not straightforward, since subjective symptoms are not highly correlated with the cartilage deterioration observed in radiographic images [9]. This fact, along with the existing uncertainty about the absolute and relative efficacy of conservative treatments, makes OA a preference-sensitive condition, in which patients’ values and preferences regarding the benefits and risks associated with different treatment options should play a decisive role when making treatment decisions [10]. A shared decision-making (SDM) process thus seems a necessary approach in this health condition, providing patients with reliable information and promoting their involvement in decision-making, according to the principles of the patient-centered care model [11].

Patient decision aids (PtDAs) are one of the most used interventions to promote SDM and help patients to decide between different treatments options [12]. They provide information on a specific health problem and its available treatments, including quantitative information about their risks and benefits, and other characteristics relevant for making a decision. They also addresses patient’s values and preferences regarding those characteristics and potential consequences, implicitly or explicitly promoting reflection about those own values/preferences. PtDAs have shown to improve patients’ knowledge about the disease and treatments, their decisional conflict, the concordance between patients’ preferences and their actual choices, or their involvement in decision-making, among other variables [13]. In the field of OA, the few existing studies have been focused on knee OA [14] or included hip patients but did not report separate analyses for them [15, 16]. Because hip OA has its own characteristics and given the existing shortages in this field, the aim of this study is to develop a PtDAs for hip OA and evaluate its effectiveness on the improvement of patients’ decisional process.

## Methods/design

**Fig. 1:**
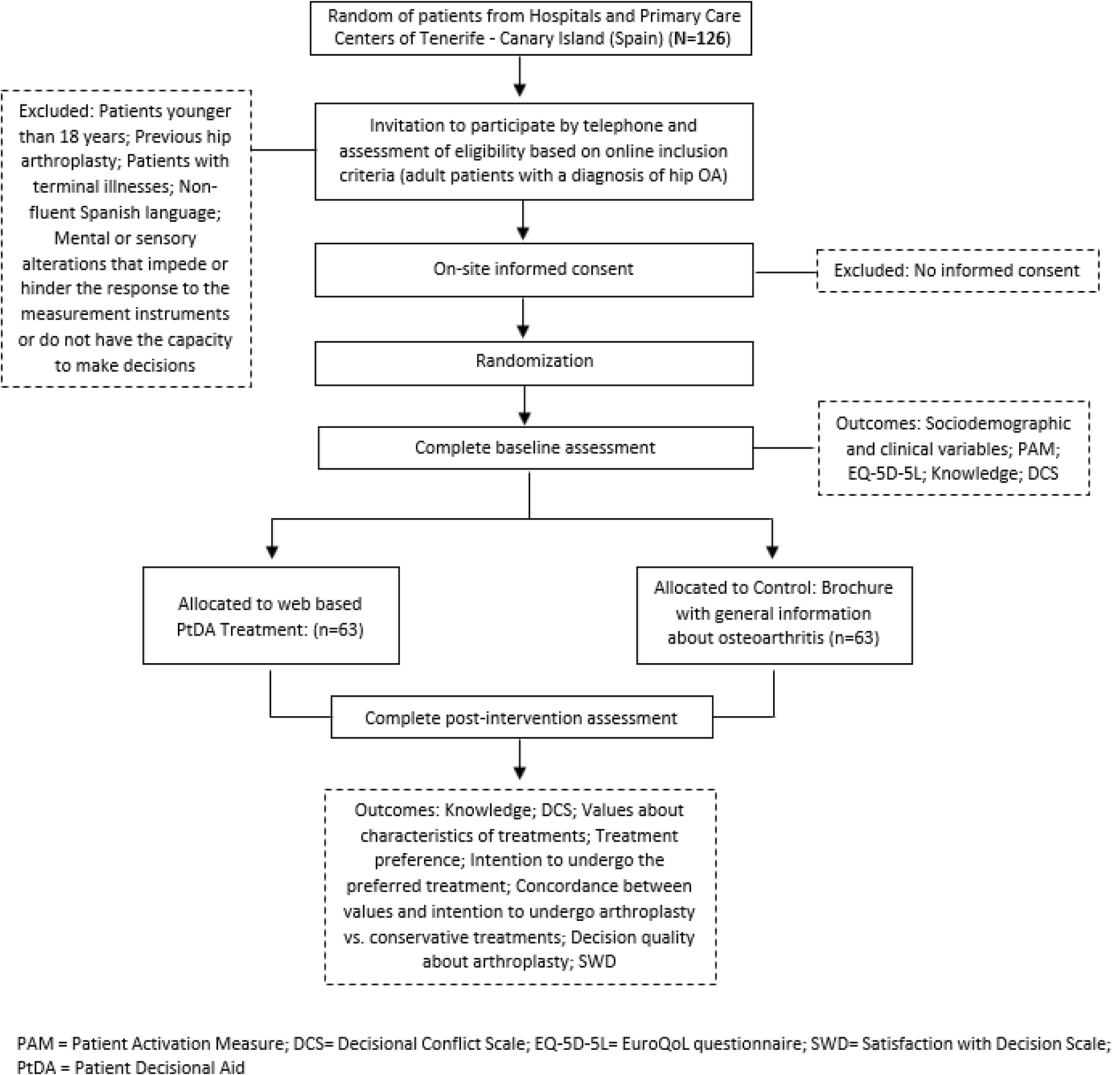
Flow of participants

**Fig. 2:**
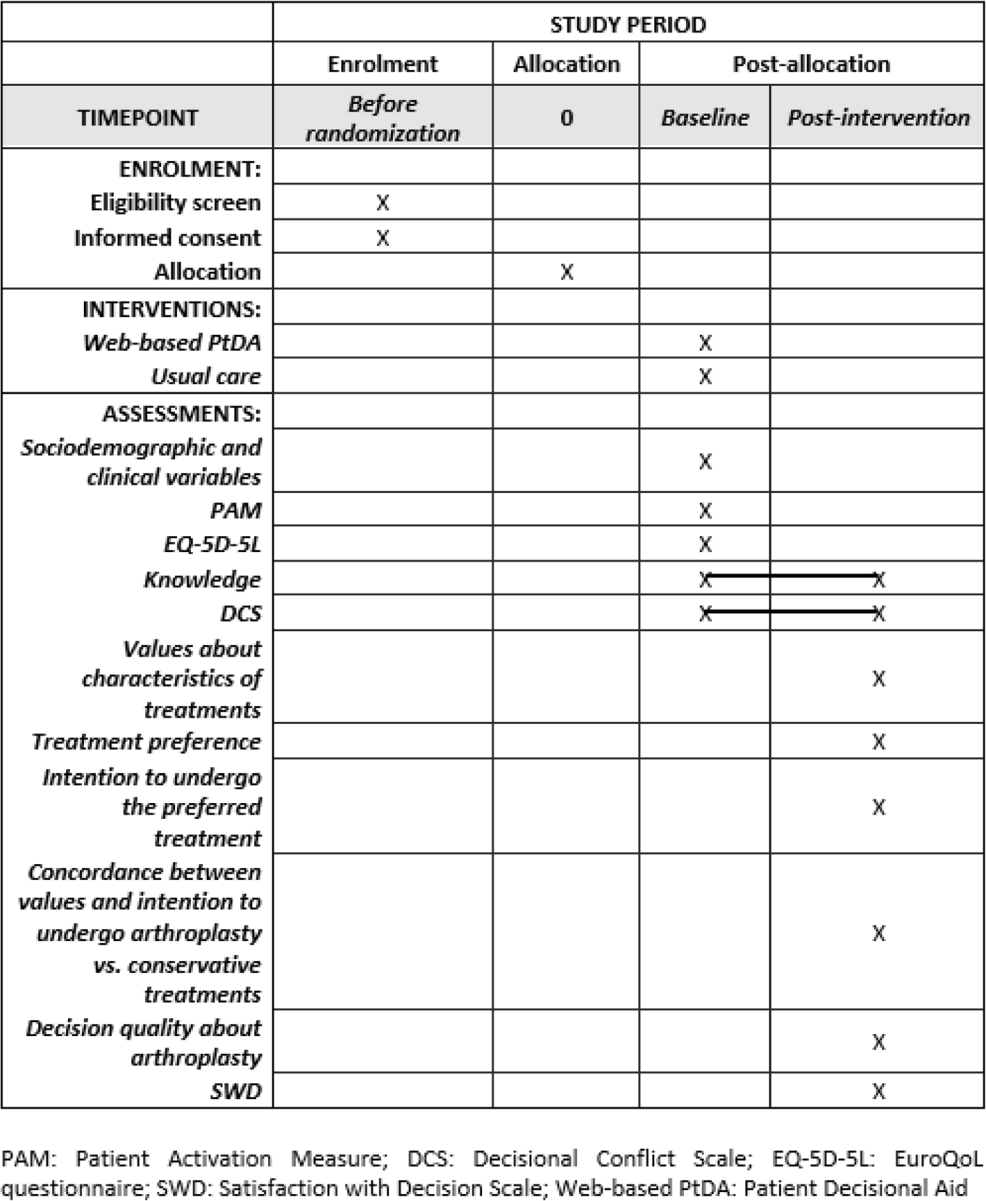
Schedule of enrolment, interventions, and assessments (SPIRIT checklist)

This clinical trial protocol follows the Standard Protocol Items: Recommendations for Interventional Trials (SPIRIT) 2013 statement [17] (see Additional file 1). Flow of participants are presented in Fig. 1 and the schedule of enrollment, intervention, and assessments is shown in Fig. 2. Any future protocol modification will be registered in the ClinicalTrials.gov database and communicated to the Scientific and Ethics Committee of the University Hospital Nuestra Señora de la Candelaria (Tenerife, Spain).

### Study design

The general design of the study consists of two phases: (1) development of a web-based PtDA for hip OA patients and (2) assessment of its effectiveness in a randomized controlled trial (RCT).

### Phase 1: Development of a web-based PtDA

The procedure for the development of the PtDA will consist of the following steps:

1. We performed two systematic reviews to identify evidence on hip OA treatments’ effectiveness (search strategy: “hip osteoarthritis AND treatment” in the title), on one side, and decision support interventions for hip and knee OA patients, on the other. We consulted the databases Medline, Embase, and WOS on January 2019. For OA treatments, we only included clinical practice guidelines and systematic reviews with meta-analysis, prioritizing those more recent with enough quality. For the second review, we included all type of empirical studies which applied a PtDA for hip and knee OA patients, with the only aim of identifying the PtDAs and try to access to its content.
2. The Project Management Group will be composed of a *scientific team* (researchers and clinical experts) with expertise in assessing the quality of the evidence presented to the Advisory Committee, and in its interpretation, and a *technical team* (web developers and designers). An Advisory Committee will also be created with 6–8 external primary and specialized care professionals (i.e., traumatology, rheumatology, physiotherapy, rehabilitation) with experience in hip OA management, and/or patient decision-making, and 3–4 individuals living with hip OA and their families and/or caregivers.
3. Evidence synthesis and prototype of the PtDA: the scientific team will analyze and discuss the evidence on the efficacy and safety of the different treatment options, based on the results of the systematic review, and a first draft of the PtDA contents and structure will be developed following the International Patients Decision Aid Standards (IPDAS) [12]. Along with the technical team, format and presentation issues will be discussed. At the end of this phase, a first version of the PtDA will be prepared.
4. Review and evaluation of contents: the PtDA will be sent to all members of the Advisory Committee. Content and presentation issues will be reviewed and refined, in an iterative process that will result in a second version of the PtDA. The review will be through virtual or face-to-face meetings, according to the availability of the members of Advisory Committee. There is not a predefined number of review cycles, and it will depend on the time schedule of the project and the consensus reached in the successive reviews.
5. Field testing: this new version of the PtDA will be tested in individual interviews with 10 hip OA patients (with and without previous arthroplasty), who will evaluate its acceptability in terms of content (quantity, clarity and usefulness of information), structure, format, and navigability. Qualitative and quantitative information will be collected. After modifying those aspects highlighted by patients in order to improve the PtDA, it will be ready to evaluate its effectiveness.

### Phase 2: Assessment of effectiveness

#### Study design and setting

A parallel, multicenter, superiority RCT will be carried out in hospitals and primary and specialty care centers of Tenerife (Spain). Patients will be randomly allocated to intervention (web-based PtDA) or control group (general information about OA).

#### Eligibility criteria

Adult patients with a diagnosis of hip OA, eligible for either surgical or non-surgical management, and with fluency to read, write, and speak Spanish will be included in the trial. Exclusion criteria will be as follows: previous hip arthroplasty, patients with terminal illnesses, be participating in other trials, or mental or sensory alterations that impede or hinder the response to the measurement instruments or do not have the capacity to make decisions.

#### Randomization and allocation concealment

Computer-based randomization with Stata® 15.0 will be performed centrally by an independent researcher (allocation rate 1:1). Randomization will be stratified by patient inclusion in waiting list for arthroplasty (yes/no). Sealed envelopes with each patient’ allocation will be prepared, which will be opened only after the patient sign the informed consent for participation in the study. The nature of the intervention makes impossible to blind patients and researchers to group allocation.

#### Recruitment and procedure

Eligible patients will be identified through the review of participating centers’ clinical charts. A member of the research team will contact them by telephone to explain the study and invite them to participate. Those who agree will complete the baseline assessment in the same phone call, and an appointment will be scheduled with a researcher at their referral primary healthcare center or hospital. There, patients will be asked to read the project information sheet, which will explain in detail what their participation will consist of, and to sign the informed consent. Then, patients allocated to the intervention group will review the PtDA accompanied by a researcher, who will give support in navigation if necessary. Then, they will complete the questionnaires assessing the outcome measures, in the same web interface. Patients in the control group will follow the same procedure, but instead of the PtDA, they will review general information about OA of the hip, knee, ankle, and foot, information included in a brochure delivered in primary care centers of the Canary Islands Health Service, which will be also presented in a web format.

#### Outcome measures

The primary outcome of this study will be decisional conflict regarding the treatment for OA, assessed with the Decisional Conflict Scale (DCS) [18], immediately after reviewing the PtDA. This is a 16-item self-reported scale widely used in PtDA research. It has 5 subscales: feeling informed, values clarity, feeling supported, uncertainty, and effectiveness. Scores are transformed to a 0–100 scale, with higher scores indicating more conflict. In previous studies in other health conditions, we have obtained good internal consistency values (0.88–0.90) [19–21]. The secondary outcomes will include:

- Knowledge of the disease and treatments: assessed with a 7-item questionnaire previously used in studies of PtDA in OA, specifically adapted for hip OA [22]. This instrument assesses patients’ objective knowledge as an outcome of the information/decisional process, and it does not measure the level of health literacy. Items have a “true/false/do not know” format. The percentage of correct responses represents the total score.
- Values about characteristics of treatments: patients will be asked to rate in a 0–10 scale the importance they attribute to different characteristics of treatment: mode of administration, improvement in pain and function, risk of mild and serious adverse effects, time until symptoms’ improvement, duration of benefits, and in the case of arthroplasty, period of rehabilitation and risk of needing surgery revision.
- Treatment preference (physiotherapy, medication, intra-articular injections, arthroplasty, not sure).
- Intention to undergo the preferred treatment: assessed with one item ranging 0 (not sure) to 5 (completely sure).
- Concordance between values and intention to undergo arthroplasty vs. conservative treatments: binary variable (yes/no) derived from the association between values and intention to undergo arthroplasty (see the “Statistical analysis” section).
- Decision quality about arthroplasty: binary variable (yes/no) defined as a combination of adequate knowledge (≥ 60% of correct responses) and concordance.
- Satisfaction with the decision-making process: assessed with the 12-item questionnaire developed by Barry et al. [23]. In a previous study with type 2 diabetes patients, we obtained a Cronbach alpha value of 0.90 [20].
- Safety: patients will be asked whether participation in the study has produced any psychological undesired outcome such as anxiety, mood changes, uncertainty, or any kind of concern.

At baseline, in the telephone call to recruit patients, assessment will include the primary outcome (DCS), as well as the following control variables: sociodemographic (i.e., age, sex, education, living and job status) and clinical data (i.e., comorbidities, affected hip, OA duration, previous and current treatments for OA, being in waiting list for arthroplasty); the Patient Activation Measure (PAM) [24]; and general health-related quality of life measured with the EuroQoL questionnaire (EQ-5D-5L) [25]. Knowledge will be assessed just before the application of the PtDA (or the OA general information in the control group). Immediately after the intervention, all the outcome measures will be assessed again.

#### Sample size calculation

Assuming type I and II errors of 0.05 and 0.20 respectively, a total of 126 patients (63 per group) are needed in order to detect a medium effect size (Cohen’s *d* = 0.5) [26] in the primary dependent variable (DCS). No losses are expected given that the post-intervention assessment will take place immediately after the application of the PtDA.

#### Statistical analysis

Data will be analyzed on an intention-to-treat basis, although given the nature of the intervention, we do not expect relevant protocol deviations. The baseline equivalence of the randomized groups will be assessed by means of Student’s *t* test and *χ*^2^ test, for continuous and categorical variables, respectively. To analyze the effect of the intervention on the primary outcome (DCS), mixed linear regression model with intervention as independent variable will be used, adjusting for DCS baseline scores and sociodemographic or clinical variables which show significant between-group differences at baseline. Center will be included as a random intercept to control for the potential intraclass correlation. The same analysis will be carried out for the remaining continuous outcomes. Treatment preference will be analyzed by means of *χ*^2^ test. Concordance between values and intention to undergo arthroplasty will be calculated by means of a method similar to the one proposed by Sepucha et al. [27]: first, a binary variable will be created, with a value of 1 for participants who prefer arthroplasty and scored 4 or 5 in intention to undergo it, and zero for the remaining patients. Second, a mixed logistic regression model will be performed with patients’ values as independent variables and intention to undergo arthroplasty (vs. other treatments) as dependent variable. Significant predictors will be retained in the model and the predicted score for each patient will be calculated. Patients with a predicted score equal or higher (lower) than 0.5 and intention to (not) undergo arthroplasty will be classified as concordant, and the remaining ones as not concordant. Finally, the effect of the DA in the number of concordant patients, as well as the number who make a quality decision, will be assessed with mixed logistic regression. For all outcomes, we will explore the interaction of the intervention with sex and education. Analyses will be performed with Stata® 15.0.

#### Data monitoring, management, and confidentiality

The project team at the Evaluation Unit at the Canary Islands Health Service (Tenerife, Spain) will serve as the data coordinating center responsible for data collection forms, coordination of data transfer, and data analysis. This is a minimal risk study and we do not expect harms derived from participation. Therefore, an independent safety data monitoring committee is not needed. Nonetheless, researches who will apply the intervention and collect outcomes data will asked for any undesired outcome and register any behavioral or verbal sign which could indicate discomfort, annoyance, or worry.

Study data will be stored on secure drives on a server’s institution (Evaluation Unit at the Canary Island Health Service) and managed according to the General Registry for the Protection of Personal Data (RGPD UE 679/2016) and Spanish Organic Law 3/2018, of December 5, on the Protection of Personal Data and Guarantee of Digital Rights. All documents that contain names or personal identifying information will be stored separately from other study data and identified by code number. Access to files will be limited to research staff involved in the study. The statistician performing the data analyses will receive depersonalized data where the participants’ identifying information will be replaced by an unrelated sequence of numbers.

#### Dissemination policy

The results of this study will be published in a peer-review journal and presented in international conferences.

## Discussion

Patient-centered care is currently advocated as the gold standard of health care, and SDM is one of its essential aspects. In order to encourage SDM, many interventions aimed at patients, health professionals, or both have been developed, to be applied or used before and/or during the clinical encounter [13, 28]. Among them, PtDAs have the strongest evidence favoring their efficacy in increasing patients’ knowledge, decreasing their decisional conflict, and in general improving the decision-making process and the quality of their health care decisions (understood as being informed and concordant with own values/preferences) [13, 28]. In the field of OA, however, the number of available PtDAs is very scarce, and published RCTs are limited and their assessed outcomes heterogeneous [13, 26, 29, 30]. Furthermore, they have included only patients with knee OA or have not reported separated data for hip patients [15, 16, 31]. Therefore, this RCT will contribute to increase the current knowledge about the effectiveness of PtDAs in the field of hip OA, with the ultimate aim of improving the quality of care offered to these patients. No physical or psychological risk is expected to result from participation in this study. Participants allocated to the PtDA group will access to updated information on the disease and the risks and benefits of the various treatments for hip OA and will be encouraged to explicitly clarify their values and preferences about those treatment options.

### Limitations and strengths

In relation to the systematic review for the development of the PtDA, it is expected to find limitations related to the coverage and completeness of the information obtained, since the bibliographic search does not guarantee the obtaining of the universe of articles. We will expand the search with consultation of other sources such as specialized websites and institutions working in this area of research.

The main limitations of the effectiveness study are the impossibility of blinding participants to treatment allocation, and the absence of follow up, which precludes the assessment of patients’ subsequent interaction with their health care providers and their actual treatment choices. We will try to mitigate the lack of blinding giving control participants a brochure with general information about OA. In the absence of data about patients’ actual decisions, we will measure the intensity of their intention to undergo their prefer treatment. On the other side, we will only collect baseline data for the primary outcome and knowledge, in order to avoid overloading patients with too many questionnaires; nonetheless, an adequate randomization and allocation process should overcome the influence of this limitation.

The strength of the study relies on its methodological rigor, beginning with the development of the PtDA based on recommended guidelines [12, 32], involving a wide number of researchers, clinical experts, and patients, and assessing its effectiveness in a RCT with adequate randomization, allocation concealment, and implementation of the intervention. Regarding the population, given the socioeconomic context (one of the less developed regions of Spain) and the advanced age of hip patients on average, it is expected to include a considerable number of patients with low education level and/or health/digital literacy, enhancing the current evidence about the effectiveness of PtDAs interventions in these vulnerable populations [33, 34]. The developed PtDA promises to be a useful resource to help patients to improve their knowledge of treatment options for hip OA, to take a more active decisional role about their health care, and to make decisions more aligned with their values and preferences. From the professionals’ perspective, it could help to improve the quality of their communication with patients, leading to a better understanding of patients’ preferences, values, and concerns. Furthermore, the use of the PtDA should reduce the variability of practice in the information/decision process, both within and across professionals. From the health system’s perspective, these tools could help to improve the efficiency of health care for OA [35, 36].

## Trial status

The trial has been registered at ClinicalTrials.gov under the identifier number NCT04241978 on 24 January 2020. The recruitment started in February 2020 and is expected to end by October 2020. This is the Version 3 (August 4, 2020).

## Supplementary information

**Additional file 1.** SPIRIT 2013 Checklist: Recommended items to address in a clinical trial protocol and related documents.

## Data Availability

The datasets used and analyzed during the current study will be available from the corresponding author on reasonable request. The PtDA will be available after the end of the study on the PyDeSalud website (https://www.pydesalud.com/).

## Abbreviations

OA: Osteoarthritis
PCC: Person-centered care
PtDA: Patient decision aid
SDM: Shared decision-making
